# Retrospective Comparison of Fludarabine in Combination With Intermediate-Dose Cytarabine Versus High-Dose Cytarabine As Consolidation Therapies for Acute Myeloid Leukemia

**DOI:** 10.1097/MD.0000000000000134

**Published:** 2014-12-12

**Authors:** Wenjun Zhang, Yi Ding, Hao Wu, Yuhua Chen, Huina Lu, Chunying Chen, Jianfei Fu, Weiguang Wang, Aibin Liang, Shanhua Zou

**Affiliations:** From the Department of Hematology, Tongji Hospital, Tongji University School of Medicine (WZ, YD, HW, YC, HL, CC, JF, AL); and Department of Hematology, Zhongshan Hospital, Fudan University (WW, SZ), Shanghai, China.

## Abstract

This retrospective study compared efficacy and safety of fludarabine combined with intermediate-dose cytarabine (FA regimen) versus high-dose cytarabine (HiDAC regimen) as consolidation therapy in acute myeloid leukemia (AML) patients who achieved complete remission.

Disease-free survival (DFS) and overall survival (OS) based on age (≥60, <60 years) and cytogenetics were evaluated from data between January 2005 and March 2013.

Total 82 patients (FA, n = 45; HiDAC, n = 37; 14–65 years) were evaluated. Five-year DFS was 32.0% and 36.2% for FA and HiDAC groups, respectively (*P* = 0.729), and 5-year OS was 39.5% and 47.8% (*P* = 0.568), respectively. Among older patients (≥60 years), 3-year DFS was 26.0% for FA group and 12.5% for HiDAC group (*P* = 0.032), and 3-year OS was 34.6% and 12.5%, respectively (*P* = 0.026). In FA group, hematological toxicities were significantly lower. FA regimen was as effective as HiDAC regimen in patients with good/intermediate cytogenetics and significantly improved DFS and OS in older patients.

## INTRODUCTION

Chemotherapeutic agents for remission induction and consolidation in acute myeloid leukemia (AML) mostly include cytarabine in combination with other anticancer drugs (eg, anthracycline or anthracenedione).^1^ Literature suggests that early studies have established the role of high-dose cytarabine for remission induction and consolidation^2^ despite the associated toxicities, especially in the elderly population.^3,4^ However, the optimal dose of cytarabine remains controversial as recent studies have challenged the role of high-dose cytarabine in both remission induction^2^ and consolidation.^3,5,6^ Regarding consolidation therapy, a variety of modifications to chemotherapeutic regimens/combinations are available in literature,^2,5,7–9^ with their primary goal being maintenance of remission or increase in disease-free survival (DFS) and overall survival (OS) with minimal toxicity. One such regimen is the FLAG regimen (fludarabine + intermediate-dose cytarabine + granulocyte colony-stimulating factor [G-CSF]) used with or without modifications in various studies for the treatment of refractory or relapsed AML,^10–16^ multilineage dysplasia (MD-AML),^17^ and consolidation therapy.^18^

In view of the available literature on the indication and applicability of the FLAG regimen, at our institute, we use a modified FLAG (FA) regimen with intermediate-dose cytarabine or high-dose cytarabine (HiDAC) as consolidation therapy. Estey et al^19^ have shown that complete response and survival rate with FA or FLAG regimens were similar in patients with newly diagnosed AML or myelodysplastic syndromes (MDS).

Furthermore, when fludarabine is used in combination with cytarabine, it potentiates the intracellular accumulation of ara-C 5′-triphosphate (ara-CTP–active metabolite of cytarabine), thereby enabling significant dose reduction of cytarabine. This is important because at the molecular level, ara-CTP saturates the phosphorylating enzymes at plasma concentrations of >10 μmol/L, thereby raising concerns regarding the use of HiDAC.^20^

The above-mentioned evidence suggests that the FA regimen with intermediate-dose cytarabine can serve as an alternative to HiDAC regimen to achieve the aim of consolidation therapy. Therefore, keeping in mind the importance of consolidation therapy in terms of efficacy, age of the patient, toxicity, and genetic heterogeneity, this retrospective study evaluated the FA regimen versus HiDAC regimen as consolidation therapy in non-M3 AML patients who achieved complete remission (CR).

## MATERIALS AND METHODS

### Study Design

This retrospective, observational study evaluated the outcome of non-M3 AML patients who achieved CR and were followed by consolidation chemotherapy with either FA or HiDAC regimen at the Tongji Hospital of Tongji University from January 2006 to March 2013. Patients who received a uniform induction regimen consisting of cytarabine (100 mg/m^2^ as a continuous intravenous infusion daily for 7 days and daunorubicin 60 mg/m^2^ intravenous push daily for 3 days) were identified. Among them, patients who achieved CR and were assigned to either FA or HiDAC regimen were evaluated. After CR, 1 or 2 cycles of additional intrathecal dexamethasone, methotrexate, and cytarabine were administered to the patients. Thereafter, patients were divided into subgroups based on their cytogenetic abnormalities, as reported in published literature.^21,22^ Patients with AML characterized by t(8; 21) or inv16 were considered to have favorable cytogenetics. Patients with –5, –7, del(5q), abnormal 3q, or complex cytogenetics—defined as the presence of at least 5 unrelated cytogenetic abnormalities—were considered to have adverse cytogenetics. The remaining patients were considered to have intermediate cytogenetics. Furthermore, these patients were not considered for hematopoietic stem cell transplantation (HSCT) because randomized studies comparing HSCT with high-dose cytarabine have failed to demonstrate any survival benefit in first remission.^23–25^ Both this study and the use of FA regimen as consolidation therapy were approved by the ethics committee of the Tongji Hospital.

### Patients

Male and female patients age between 14 and 65 years with non-M3 AML who achieved CR with 1 or 2 cycles of induction chemotherapy and were subsequently treated with FA or HiDAC regimen were included. Patients with severe cardiopulmonary insufficiency were excluded.

### Treatment

Consolidation therapy with the FA regimen consisted of fludarabine at a dose of 30 mg/m^2^ followed 4 h later by cytarabine at a dose of 1000 mg/m^2^ over 3 h daily for 3 days. Consolidation therapy with the HiDAC regimen consisted of cytarabine administered at a dose of 2000 mg/m^2^ through 3 h intravenous infusion at 12-h intervals on days 1, 2, and 3. In addition, dexamethasone eye drops were used as prophylaxis. Best supportive care included the administration of antibiotics and red blood cells and platelet transfusions, when indicated. To minimize the risk of transfusion-associated graft-versus-host disease, patients who required blood transfusion in the FA group were transfused with irradiated blood only.

### Definitions and Evaluation of Response

CR was defined as the reconstitution of normal cellular bone marrow with <5% blast cells along with a peripheral blood neutrophil count >1.0 × 10^9^/L, a platelet count >100 × 10^9^/L, and no evidence of extramedullary leukemia. Relapse was defined as the presence of at least one of the following: reappearance of leukemic blasts in peripheral blood, recurrence of >5% blasts in bone marrow, and appearance of extramedullary leukemia. DFS was defined as the time from achievement of CR to disease recurrence or death from any cause. OS was determined as the time interval from the date of diagnosis to the date of last follow-up or death.

### Assessments

Bone marrow examination was performed to confirm CR in both groups before each consolidation therapy and at the end of all consolidation therapies. DFS and OS were evaluated among older (≥60 years) and younger (<60 years) patients and those distributed in the 3 cytogenetic subgroups. Safety assessments included adverse event profile and hematological and nonhematological toxicity, which were recorded according to the World Health Organization (WHO) criteria.^26^

### Statistical Analysis

Descriptive statistics were used to assess patient demographics, disease characteristics, and related covariates of interest. Categorical variables such as gender, French–American–British (FAB) subgroup, cytogenetics analysis, time of induction to first remission, toxic grade, and support care were summarized with counts and percentages. Continuous variables such as age at diagnosis, initial white blood cell counts, and follow-up duration were summarized as median and range, as necessary. Chi-square test was used to assess the association between the categorical variables and nonparametric test was used to compare continuous variables between the 2 regimens. DFS and OS were calculated by the Kaplan–Meier method,^3^ and the log-rank test was applied to calculate the significance of differences between survival curves. The *P* value for all statistical comparisons was two-tailed and differences were considered to be statistically significant when the *P* value was <0.05. Statistical analyses were performed using SPSS version 19.0 (SPSS Inc, Chicago, IL) and GraphPad Prism version 5.01 software for Windows (GraphPad Software, San Diego, CA).

## RESULTS

### Patient Characteristics

A total of 82 patients who met the inclusion criteria were identified. Of these, 45 patients (age range: 14–65 years) received the FA regimen and 37 patients (18–65 years) received the HiDAC regimen. There was a similar distribution of patients with regard to gender, age, initial white blood cells, FAB classification, cytogenetics, and courses of induction therapy to time of induction to first remission between the 2 treatment groups (*P* > 0.05) (Table 1). A similar patient distribution was noted between the 3 cytogenetic subgroups in the 2 treatment groups (*P* > 0.05) (Table 2 ). All patients identified were evaluated, and no dropout, loss to follow-up, or noncompliance was reported in the study.

**TABLE 1:**
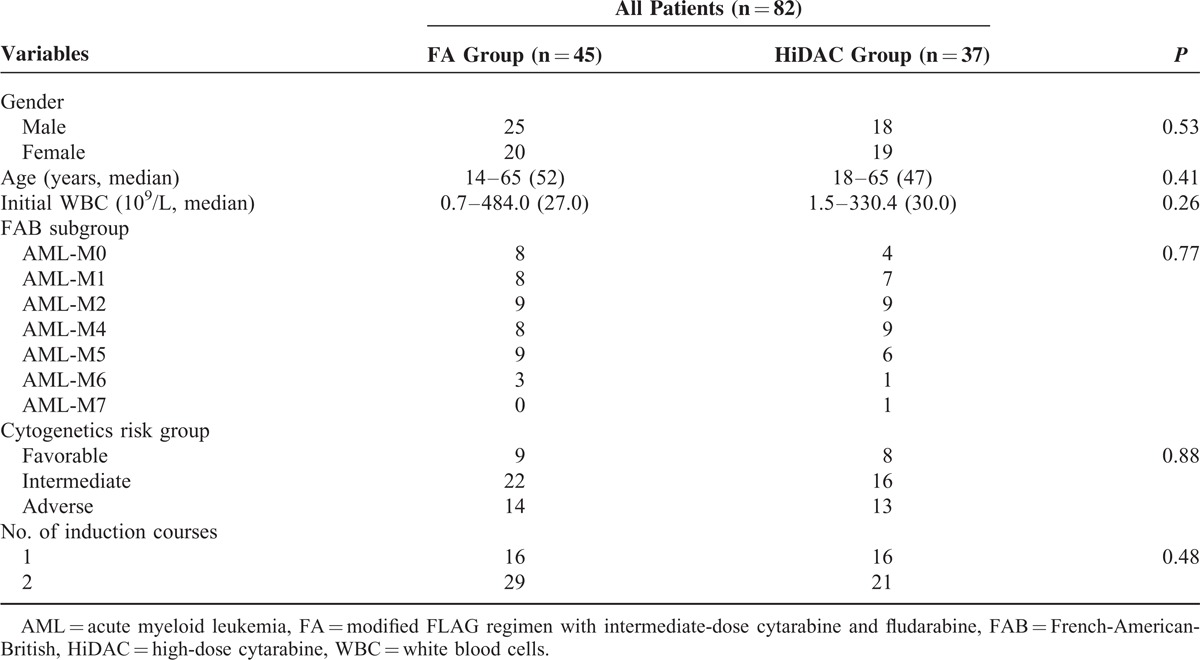
Baseline Characteristics of the Patients

**TABLE 2:**
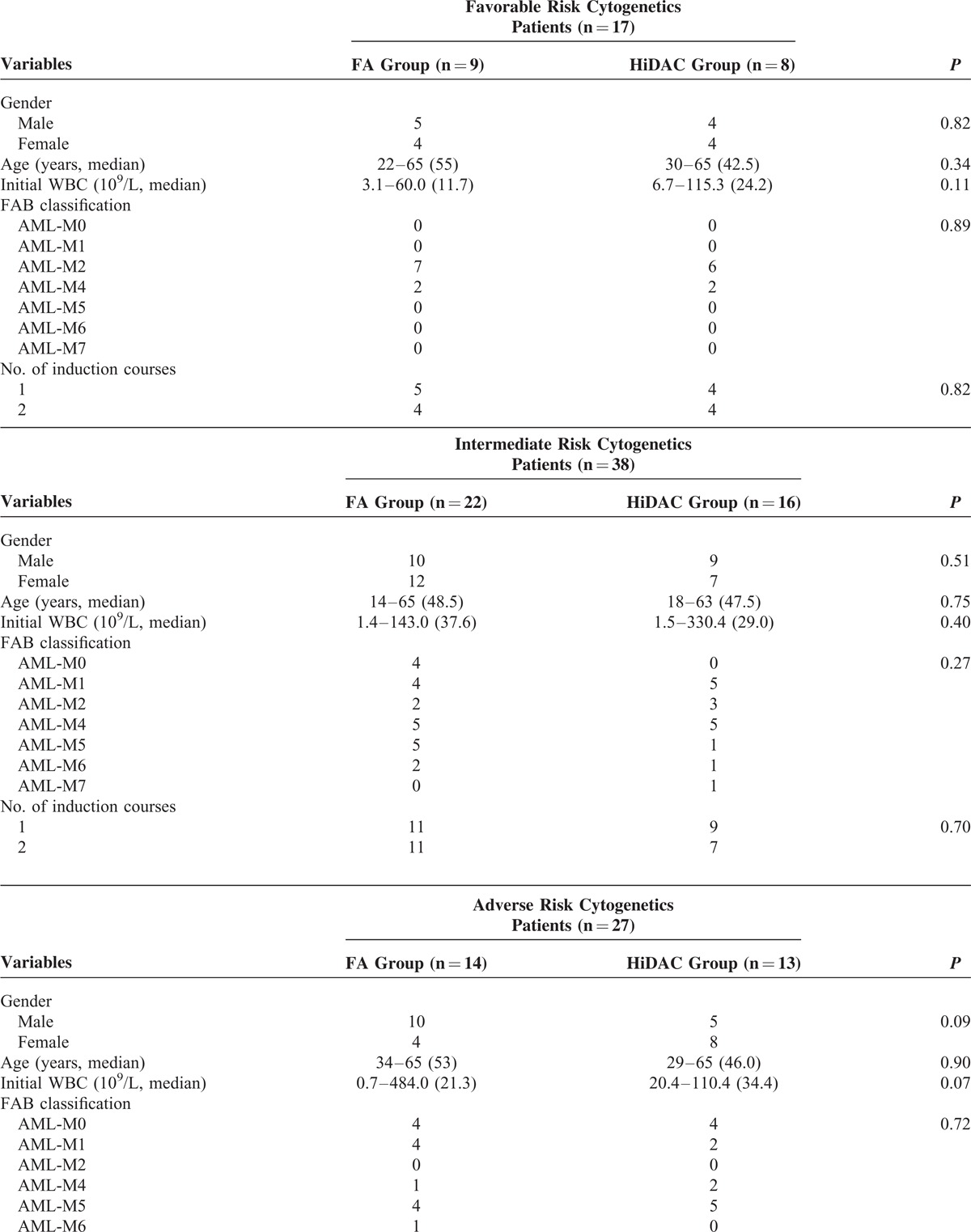
Baseline Characteristics of the Patients by Cytogenetics Subgroups

**TABLE 2 (Continued):**
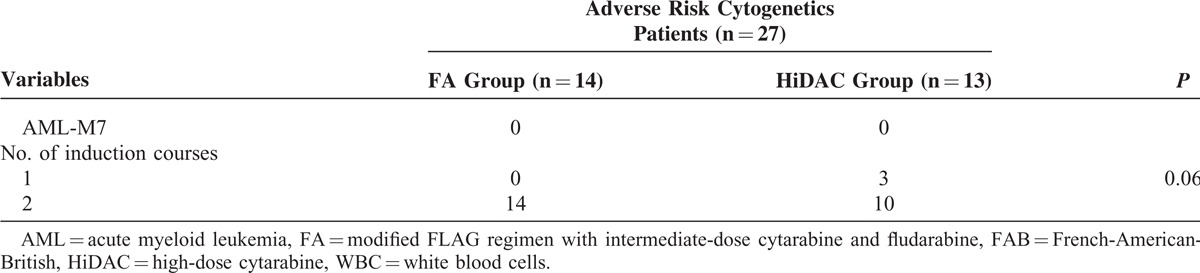
Baseline Characteristics of the Patients by Cytogenetics Subgroups

### Outcome of Consolidation

**FIGURE 1:**
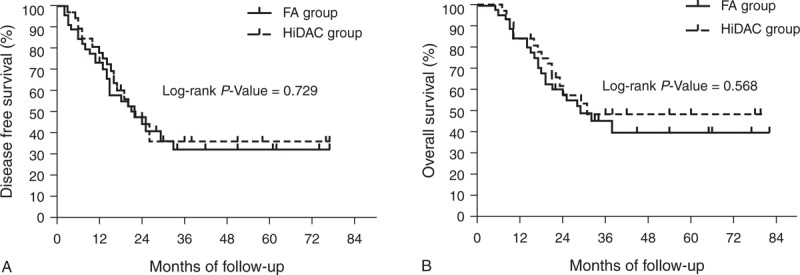
Probability of DFS and OS by treatment arm for all patients with AML (A) Predicted 5-year DFS was 32.0% for the FA group (n = 45; solid line) and 36.2% for the HiDAC group (n = 37; dotted line; *P* = 0.729). (B) Predicted 5-year OS was 39.5% for the FA group (n = 45; solid line) and 47.8% for the HiDAC group (n = 37; dotted line; *P* = 0.568). AML = acute myeloid leukemia, DFS = disease-free survival, FA = modified FLAG regimen with intermediate-dose cytarabine and fludarabine, HiDAC = high-dose cytarabine, OS = overall survival.

The median follow-up of all the surviving patients was 29 months (range: 9–82 months). At the 5-year follow-up, 4 patients each in the FA and HiDAC groups remained alive. Survival curves for all patients in the FA group versus HiDAC group are shown in Figure 1. Furthermore, the 5-year DFS was 32.0% and 36.2% (*P* *=* 0.729, Figure 1A) and the 5-year OS was 39.5% and 47.8% (*P* = 0.568, Figure 1B) for the FA and HiDAC groups, respectively.

**FIGURE 2:**
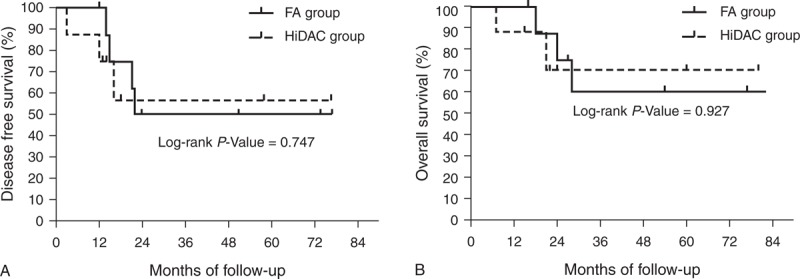
Probability of DFS and OS by treatment arm for the favorable cytogenetic risk group (A) Predicted 5-year DFS was 50.0% for the FA group (n = 9; solid line) and 56.3% for the HiDAC group (n = 8; dotted line; *P* = 0.747). (B) Predicted 5-year OS was 60.0% for the FA group (n = 9; solid line) and 70.0% for the HiDAC group (n = 8; dotted line; *P* = 0.927). DFS = disease-free survival, FA = modified FLAG regimen with intermediate-dose cytarabine and fludarabine, HiDAC = high-dose cytarabine, OS = overall survival.

**FIGURE 3:**
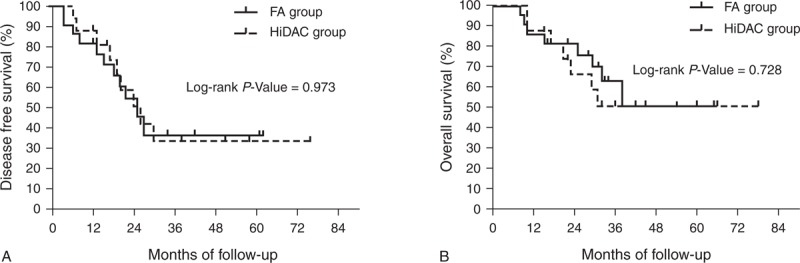
Probability of DFS and OS by treatment arm for the intermediate cytogenetic risk group (A) Predicted 5-year DFS was 36.3% for the FA group (n = 22; solid line) and 33.6% for the HiDAC group (n = 16; dotted line; *P* = 0.973). (B) Predicted 5-year OS was 50.5% for the FA group (n = 22; solid line) and 50.8% for the HiDAC group (n = 16; dotted line; *P* = 0.728). DFS = disease-free survival, FA = modified FLAG regimen with intermediate-dose cytarabine and fludarabine, HiDAC = high-dose cytarabine, OS = overall survival.

**FIGURE 4:**
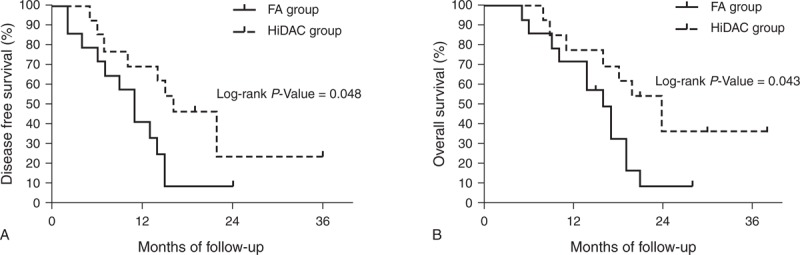
Probability of DFS and OS by treatment arm for the adverse cytogenetic risk group (A) Predicted 2-year DFS was 8.2% for the FA group (n = 14; solid line) and 23.1% for the HiDAC group (n = 13; dotted line; *P* = 0.048). (B) Predicted 2-year OS was 8.2% for the FA group (n = 14; solid line) and 35.9% for the HiDAC group (n = 13; dotted line; *P* = 0.043). DFS = disease-free survival, FA = modified FLAG regimen with intermediate-dose cytarabine and fludarabine, HiDAC = high-dose cytarabine, OS = overall survival.

**FIGURE 5:**
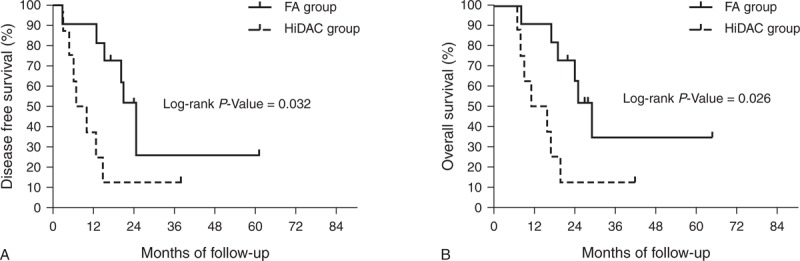
Probability of DFS and OS by treatment arm for older patients (A) Predicted 3-year DFS was 26.0% for the FA group (n = 11; solid line) and 12.5% for the HiDAC group (n = 8; dotted line; *P* = 0.032). (B) Predicted 3-year OS was 34.6% for the FA group (n = 11; solid line) and 12.5% for the HiDAC group (n = 8; dotted line; *P* = 0.026). DFS = disease-free survival, FA = modified FLAG regimen with intermediate-dose cytarabine and fludarabine, HiDAC = high-dose cytarabine, OS = overall survival.

In patients with favorable cytogenetics, the 5-year DFS was 50.0% and 56.3% (*P* = 0.747) and the 5-year OS was 60.0% and 70.0% (*P* = 0.927) for the FA and HiDAC groups, respectively (Figure 2, which shows 5-year DFS [Figure 2A] and 5-year OS [Figure 2B]). In patients with intermediate cytogenetics, the 5-year DFS was 36.3% and 33.6% (*P* = 0.973) and the 5-year OS was 50.5% and 50.8% (*P* = 0.728) for the FA and HiDAC groups, respectively (Figure 3, which shows 5-year DFS [Figure 3A] and 5-year OS [Figure 3B]). In patients with adverse cytogenetics, the 2-year DFS was 8.2% and 23.1% (*P* = 0.048) and the 2-year OS was 8.2% and 35.9% (*P* = 0.043) for the FA and HiDAC groups, respectively (Figure 4, which shows 2-year DFS [Figure 4A] and 2-year OS [Figure 4B]).

**FIGURE 6:**
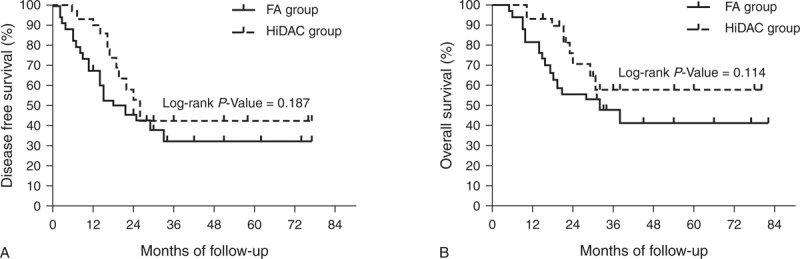
Probability of DFS and OS by treatment arm for younger patients (A) Predicted 5-year DFS was 32.0% for the FA group (n = 34; solid line) and 42.2% for the HiDAC group (n = 29; dotted line; *P* = 0.187). (B) Predicted 5-year OS was 41.2% for the FA group (n = 34; solid line) and 57.4% for the HiDAC group (n = 29; dotted line; *P* = 0.114). DFS = disease-free survival, FA = modified FLAG regimen with intermediate-dose cytarabine and fludarabine, HiDAC = high-dose cytarabine, OS = overall survival.

Among older patients (≥60 years), the 3-year DFS was 26.0% and 12.5% (*P* = 0.032) and the 3-year OS was 34.6% and 12.5% (*P* = 0.026) for the FA and HiDAC groups, respectively (Figure 5, which shows 3-year DFS [Figure 5A] and 3-year OS [Figure 5B]). The clinical characteristics and outcomes of older patients are shown in Table 3. Among the younger patients (<60 years), the 5-year DFS was 32.0% and 42.2% (*P *= 0.187) and the 5-year OS was 41.2% and 57.4% (*P* = 0.114) for the FA and HiDAC groups, respectively (Figure 6, which shows 5-year DFS [Figure 6A] and 5-year OS [Figure 6B]).

**TABLE 3:**
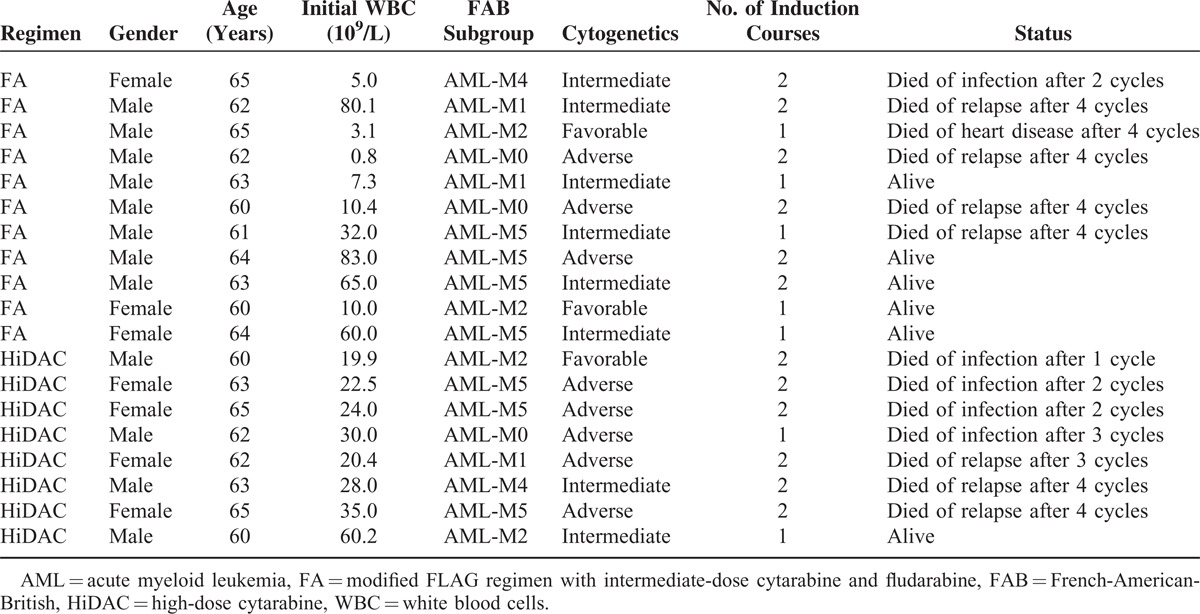
Characteristics and Outcomes of Older Patients

### Hematological Toxicity of Consolidation Therapy

A total of 168 and 137 chemotherapy courses were given to 45 and 37 patients in the FA and HiDAC treatment arms, respectively. The tolerance to FA and HiDAC regimens (including relapse and death) in terms of number and age (<60 years and ≥60 years) of patients who received 4 courses of chemotherapy is presented in Table 4.

**TABLE 4:**
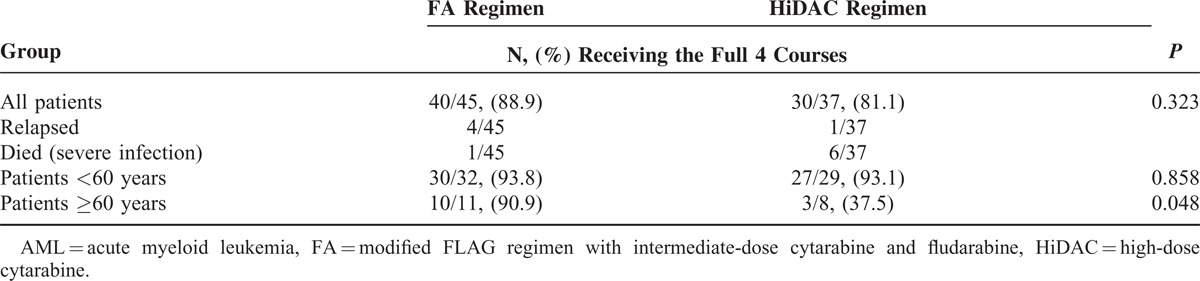
Tolerance of Modified FLAG Regimen with Intermediate-Dose Cytarabine and Fludarabine, and High-Dose Cytarabine Regimen Among Patients With AML

Hematological toxicity of consolidation chemotherapy regimen is presented in Table 5. Compared with the FA group, the HiDAC group was associated with more life-threatening anemia (WHO grade 4; 21.4% versus 79.6%, *P* < 0.001), leukopenia (58.9% versus 86.1%, *P* < 0.001), neutropenia (74.4% versus 95.6%, *P* < 0.001), and thrombocytopenia (76.2% versus 96.4%, *P *<* *0.001). A significantly higher frequency of severe infections was documented during severe neutropenia in the HiDAC group compared with the FA group (35.8% versus 24.4%, *P* = 0.03). Furthermore, the white blood cell recovery was higher than 2.0 × 10^9^/L, with a significantly shorter median time of 18.0 days (95% confidence interval [CI]: 17.9–20.4) in the FA group versus 23.0 days (95% CI: 22.6–25.0) in the HiDAC group (*P* < 0.001). Red blood cell transfusions were required in 11.3% and 43.8% of chemotherapy courses in the FA and HiDAC groups, respectively (*P* < 0.001). Platelet transfusions were required in 61.9% and 88.3% of chemotherapy courses in the FA and HiDAC groups, respectively (*P* < 0.001). Overall, the FA regimen was less toxic than the HiDAC regimen.

**TABLE 5:**
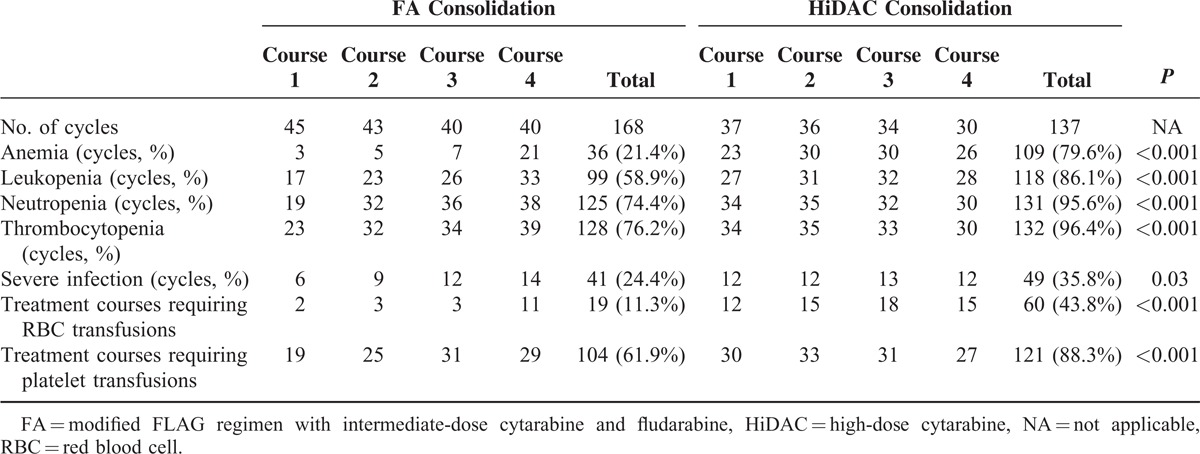
Hematological Toxicity of Consolidation and Support Care

### Nonhematological Toxicity of Consolidation Therapy and Support Care

A total of 82 patients in both groups experienced grade 1 and 2 nonhematological toxicities, including nausea, vomiting, and baldness (data not shown). Fifteen patients reported liver dysfunction, which recovered after treatment with hepatoprotective agents. Twenty-six patients in the FA group received antiviral prophylaxis. Fifteen of 19 (78.9%) patients who did not receive prophylaxis had herpes zoster virus infection compared with 8 of 26 (30.8%) patients who received antivirus prophylaxis. There was a significant difference between those who received and did not receive antivirus prophylaxis (*P* = 0.001).

## DISCUSSION

This retrospective study evaluated the FA regimen versus the HiDAC regimen as consolidation therapy in non-M3 AML patients who achieved CR. The FA regimen was as effective as the HiDAC regimen for consolidation in terms of DFS and OS in AML patients with good or intermediate cytogenetics but with less hematological toxicities. Furthermore, the FA regimen also significantly improved DFS and OS in older patients.

Two decades ago, the Cancer and Leukemia Group B Study (CALGB) trial^3^ demonstrated that HiDAC was superior to standard-dose cytarabine in improving DFS, making HiDAC therapy the standard consolidation regimen for patients <60 years. However, subsequently, numerous studies challenged the role of HiDAC in the consolidation of AML. The Australasian Leukemia and Lymphoma Group AML trial number 7 protocol indicated that HiDAC did not confer additional benefits compared with the conventional dose of cytarabine for consolidation.^27^ The CALGB-9222 study^28^ also showed no difference in DFS and OS between the HiDAC group and the intensified sequential multiagent chemotherapy group. Similarly, the JALSG AML201 study^5^ showed that HiDAC was not superior to multiagent chemotherapy in improving the DFS and OS. The Leukemia Working Group of the Dutch–Belgian Cooperative Trial Group for Hemato-Oncology (HOVON) and the Swiss Group for Clinical Cancer Research (SAKK) study revealed that no significant differences in event-free survival (EFS) or OS were noted between intermediate-dose cytosine arabinoside and HiDAC.^29^ Similarly, our study showed that there was no statistically significant difference in DFS or OS between the FA group and the HiDAC group. HiDAC regimen showed no extra benefits in improving the survival of AML patients compared with FA regimen.

Cytogenetics is considered one of the most valuable prognostic determinants in adult AML patients^22^ as a specific subgroup could benefit from the HiDAC regimen.^30^ Chromosomal abnormalities with favorable cytogenetics targeting the core binding factor (CBF), including t(8; 21)—morphologically associated with the FAB AML-M2—and inv16—associated with AML-M4Eo—are reported to respond most effectively to the HiDAC regimen.^5,30^ In our study, the distribution of patients between favorable, intermediate, and adverse cytogenetics was similar to that reported in literature for a similar Chinese population with AML.^31^ Our study showed that the postremission 5-year DFS of CBF leukemia treated with HiDAC regimen was 56.3%, which is similar to that in the JALSG AML201 study (57%)^5^ and slightly more than that reported by Bloomfield et al^30^ (50%). One possible explanation for the difference is that some patients with CBF abnormality have *KIT* mutations, which confer higher relapse risk on CBF AML. Furthermore, a high mutation rate of *c-KIT* is reported among Asian patients with t(8; 21) from China^32^ and Japan^33^. This high mutation rate of *c-KIT* might result in lower DFS. Unfortunately, our study did not perform the screening of mutations in the entire coding region of *c-KIT* gene during the time the 2 cohorts were treated. In our study, there was no significant difference in DFS or OS between patients with t(8; 21) (FA, n = 7; HiDAC, n = 6) and inv16 (FA, n = 2; HiDAC, n = 2) in the 2 treatment groups (Figure 2A and B). These results were consistent with those reported by Borthakur et al.^34^ Although there was no significant difference in DFS or OS for patients with intermediate cytogenetics, patients with adverse cytogenetics reported a higher 2-year DFS and OS in the HiDAC group compared with the FA group. This suggests that the HiDAC regimen was superior to the FA regimen in improving the prognosis of patients with adverse cytogenetics and may be recommended in patients without a human leukocyte antigen-matched donor.

Pharmacokinetic studies have shown that the accumulation of ara-CTP in leukemic cells at a dose of 3000 mg/m^2^ cytarabine is far above the saturating concentrations.^35,36^ The dose of cytarabine is clinically relevant because HiDAC is associated with life-threatening toxic effects (WHO grade 4), which are serious and appear to be cumulative. In our study, the HiDAC regimen (4 cycles of cumulative cytarabine dose 48 g/m^2^) resulted in higher incidence of life-threatening hematocytopenia, longer duration of neutropenia, higher frequency of documented infections, and higher number of transfusions compared with the FA regimen (only 12 g/m^2^); however, it was equally efficacious. The possible mechanism is that the active metabolite of fludarabine, F-ara-ATP, inhibits the enzyme ribonucleotide reductase and lowers intracellular deoxyribonucleoside biphosphate pools, thereby resulting in increased accumulation of intracellular ara-CTP in the FA regimen. This benefit was more evident among older patients^37^ who are unable to tolerate the severe toxic effect of HiDAC (therefore, HiDAC is only recommended for younger patients [<60 years]).^3^ In our study, out of the 8 patients age between 60 and 65 years and treated with HiDAC, 4 (50%) died of myelosuppression-induced severe infection. However, no infection-related deaths were reported among the 11 patients age between 60 and 65 years in the FA group. Moreover, the 3-year OS in the FA group was 34.6%, thus making the FA regimen a possible alternative optimized regimen for elderly AML patients. However, it should also be noted that there is no evidence from prospective studies that intermediate- or high-dose cytarabine (HiDAC, 0.5–3 g/m^2^) is superior to conventional-dose consolidation chemotherapy in older patients,^3,38^ but there are studies in favor of low-intensity prolonged consolidation in elderly patients.^39,40^

Furthermore, van Prooijen et al^41,42^ showed that the intermediate-dose cytarabine could not efficiently prevent relapse of the central nervous system (CNS) leukemia because of low detectable cerebrospinal fluid concentration (0.5 μmol/L [far below 10 μmol/L, achieved by cytarabine]) of cytarabine, requiring additional administration of intrathecal methotrexate and cytarabine to eliminate the remaining blast cells in the CNS. In our study, all patients achieving CR received 1 or 2 cycles of additional intrathecal chemotherapy before consolidation therapy. We report 1 patient in the FA group with CNS relapse, with no report of severe neurologic toxicity in both treatment groups. Although herpes virus infections occur frequently following treatment with fludarabine,^43^ we observed 23 patients with herpes virus infection in FA-treated groups. We report herpes zoster infection rates of 78.9% without antivirus prophylaxis and 30.8% after prophylaxis with acyclovir, suggesting that antiviral prophylaxis should be considered in the FA regimen.

Our study limitations included retrospective design, age range of elderly patients between 60 and 65 years, and unknown mutational status of numerous genes relevant in AML (few examples include neuroblastoma RAS viral [*v-ras*] oncogene homolog [*NRAS*], fms-related tyrosine kinase 3 [*FLT3*], nucleophosmin [nucleolar phosphoprotein B23, numatrin] [*NPM1*], the runt-related transcription factor 1 [*RUNX1*], v-kit Hardy-Zuckerman 4 feline sarcoma viral oncogene homolog [*KIT*], CCAAT/enhancer binding protein [C/EBP], α [CEPBA], mixed-lineage leukemia [trithorax homolog, *Drosophila*] [*MLL*], and the tet oncogene family member 2 [*TET2*]). Further, for more refined risk stratification, the influence of comorbidity was not assessed, and flow cytometry and polymerase chain reaction-based assays were not used for monitoring therapy effectiveness. However, it must be noted that the clinical utility of these assays has not been demonstrated in large prospective studies.

In conclusion, FA was an effective consolidation regimen with less hematological toxicity compared with HiDAC in AML patients with good or intermediate cytogenetic risks. This was particularly evident in patients age between 60 and 65 years, for whom the FA regimen might be an optimizing therapy. In contrast, consolidation therapy with HiDAC was more appropriate for patients with poor cytogenetic risk profile.
